# Evaluation of Maternal-Neonatal Outcomes in Vaginal Birth After Cesarean Delivery Referred to Maternity of Academic Hospitals 

**Published:** 2016-12

**Authors:** Masoumeh Mirteymouri, Sedigheh Ayati, Leyla Pourali, Mahboubeh Mahmoodinia, Maliheh Mahmoodinia

**Affiliations:** 1 Department of Gynecology, Women’s Health Research Center, Ghaem Hospital, Mashhad University of Medical Sciences, Mashhad, Iran; 2 Department of Gynecology, Research Center for Patient Safety, Mashhad University of Medical Sciences, Mashhad, Iran

**Keywords:** Vaginal Birth, Cesarean Section, Maternal Complications, Neonatal Complications

## Abstract

**Objective:** To evaluate the maternal and neonatal complications of vaginal birth after cesarean section (VBAC).

**Materials and methods:** This cross sectional study was conducted in Mashhad University of medical sciences. Eighty women with previous cesarean section who were candidate for VBAC were enrolled the study. Patients were followed up for 6 weeks after delivery. The complication of VBAC was compared between successful or unsuccessful VBAC cases. Data was analyzed by SPSS version 16.

**Results:** VBAC success rate was 91%. Post-partumhemorrhage occurred in 2.7% of woman with successful VBAC and 1.3% of CS cases. Maternal and neonatal death did not happen during our study, and none of our cases experienced uterine rupture, dystocia and neonatal tachypnea. Neonatal complications include NICU admission and neonatal resuscitation frequency in VBAC and CS were 6.8% and 57.1%, respectively (p = 0.002). Birth weight of neonates in successful VBAC was 2940 ± 768 grams and 3764 ± 254 grams in unsuccessful VBAC and this difference was significant (p = 0.007). Mean maternal admission duration in VBAC and CS were 1 ± 0.1 days and 2 ± 0.4 days (p < 0.001). Successful breastfeeding rate were higher in VBAC patients (95.8%) in comparison with CS (42.9%) and this difference was statistically significant (p = 0.002).

**Conclusion:** Our results revealed that VBAC can be considered as a safe maternal and neonatal delivery method in patients with past CS women.

## Introduction

The number of women giving birth by cesarean section (CS) has increased in recent decades (1). Although CS can reduce childbirth-related complications in complicated pregnancies, it might be more risky and expensive in normal ones (2). 

It is estimated that the rate of CS be quite high in various part of Iran. Its prevalence in urban regions reported between 38% and 48%, and this rate is 74% in the capital of Iran (Tehran) (3). The expected standard rate of CS in developing countries is estimated about 15% (4). 

One major complication of preferred CS is its influence on delivery method of the next pregnancy. For a long time, it was thought women with CS history should undergo cesarean for the all next deliveries. The main cause of C-section in our country is past CS, while in developed countries vaginal birth after cesarean section (VBAC) is considered as an alternative method for women with CS history (5). 

It is estimated that 60%-80% of women with c-section history can have vaginal delivery. In these cases, CS possibility should be considered if fetal heart rate decreases or lack of progress in labor. So, VBAC should be performed in equipped hospitals with the supervision of an obstetrician (6). Most women are not aware of the possibility of VBAC, or affectedbyfactors like fears and anxiety about maternal and neonatal complication of VBAC. Therefore, this issue can be considered in perinatal education (7). 

VBAC is convenient in women want to have more children. Other advantages of VBAC include lower infection rate, shorter hospital admission duration and etc (8, 9). VBAC main complication is uterine rupture (its rate is estimated lower than 1%) and other abdominopelvic organs damage. VBAC should be performed in women with previous transverse incision (10- 12). 

This study aimed to evaluate the maternal and neonatal complications of VBAC.

## Materials and methods

This cross sectional study was supervised by the ethical committee of Mashhad University of medical sciences in 2014- 2015. The ethical code number was IR.MUMS.REC.1393.174, and informed consent as Helsinki tent was obtained from all participants.

Pregnant women at term with history of one CS who decided to have VBAC, were enrolled the study. Study design was described for all participants and written consent was obtained from them. Sample size was estimated at list 50 cases regard to previous studies (7). Study was conducted in three referral academic hospitals in Mashhad. 

Pregnant women who were candidate for VBAC, without need for induction were enrolled the study. Women were excluded with previous classic incision, history f uterine surgery such as myomectomy, fetal or maternal indication for CS, uterine anomalies, macrosomia and more than one previous CS. Demographic data (such as mother age, gestational age, birth weight and etc) were recorded in a checklist. Previous C-section indication, after birth complications, and follow up finding during the first 6 weeks after delivery were recorded. All deliveries were conducted under the supervision of an experienced obstetrician in academic hospitals. CS equipments were ready in all deliveries and mater and fetus condition were controlled closely during VBAC.

Data were coded and entered the Statistical Package for the Social Sciences (SPSS) version 16. Qualitative variable compared with chi-square and fisher exact tests. Quantitative data were tested for normality (Kolmogorov-smirnovtest), and then differences were analyzed by T-test, wilcoxon and Mann-Whitney tests. Significance level was considered as 0.05 in all tests.

## Results

Eighty women were enrolled the study during this time. 2 fetuses died before term due to trauma. The most common causes of previous CS were lack of progress (23 cases), fetal distress (19 cases), abnormal presentations (17 mothers), vaginal hemorrhage (17 cases), meconium (11 cases), macrosomia (17 cases) and one twin pregnancy. 63 mothers (78.7%) were healthy, 7 cases (8.7%) had hypertension, frequency of diabetes mellitus, cardiac, liver diseases, hematologic diseases were 2.9%, 2.9%, 2.9% and 5.8%, respectively. 

78 mothers (97.5%) were referred regularly for prenatal care. Only 6 women (7.5%) participated in health training course during pregnancy. All fetus presentation was cephalic. Successful VBAC rate was 91.2% (73 cases). 7 cases (8.8%) underwent CS due to lack of progress (5 cases) and fetal distress (2 cases). In table 1 demographic characteristics of successful and unsuccessful VBAC were compared. 

7 neonates (8.8%) needed resuscitation in delivery room. Neonatal and maternal complications of successful and unsuccessful VBAC were compared in table 2.

## Discussion

Vaginal delivery can be an alternative choice for women with history of cesarean section (13-15). Many mothers d not aware of this possibility and think they have to undergo CS because of previous C-section.

**Table 1:** Compression of demographic characteristics between successful and unsuccessful VBAC

		Mean ±SD	P-value
**Mather age (yrs)**	**Successful VBAC**	**28 ** **±4**	**0.481** *
	**Unsuccessful VBAC**	**27 ** **±3**	
**BMI (kg/m2)**	**Successful VBAC**	**24 ** **±1**	**0.132** *
	**Unsuccessful VBAC**	**25** **±** **1**	
**Birth weight (gr)**	**Successful VBAC**	**2949 ** **±** ** 768**	**0.007** *
	**Unsuccessful VBAC**	**3764** **±** **254**	
**Hospital stay (days)**	**Successful VBAC**	**1 ** **±** ** 0.1**	**< 0.001** **
	**Unsuccessful VBAC**	**2** **±** **0.4**	
**Number of previous vaginal delivery**	**Successful VBAC**	**1** **±** **1**	**0.517** *
	**Unsuccessful VBAC**	**1**	

* Mann Whitney;

** t-test

On the other hand there are not enough evidences about VBAC safety for mother and child (16-19). So, the aim of this study was t evaluate VBAC success rate and complications in a multi centric study. 

VBAC success rate was 91% in our study, near to Frass (87%) and Bangal (85%) reports (20, 21). Damle estimated this rate between 80% and 85%, also (22). Melamedstudy result in 61% successful VBAC (14), this low rate might occur due to previous CS causes, it seems that VBAC is more difficult and impossible in cases with a history of lack of progress. Some studies revealed that VBAC success rate increases in women with CS history due to malpresentation and fetal distress in comparison with lack of progress (18).Knight success rate was 63%, and this lower rate happened because of higher birth weight (10). Phelan showed that VBAC is associated with fetus weight (23). Our findings confirmed this idea; birth weight of neonates delivered by VBAC was 600 grams lower than CS. 

The main causes of VBAC failure were lack of progress (71%) and fetal distress (29%). Melamed showed that lack of progress is associated with unsuccessful VBAC (14). 

Only 2 mothers experienced VBAC complications as post partum hemorrhage (2.7%) and one of them needed transfusion. Post partum hemorrhage occurred in 2.2% of Melamed study population (14). Transfusion rate was 2% in Frass study (24). These findings confirmed the low incidence of hemorrhagic events required transfusion in women undergo VBAC. 

None of our cases had uterine rupture, as same as Melamed (14). In Ramirez report, uterine rupture frequency was 2.4%, and most cases occurred after induction (24). It seems that selecting women for VBAC is very important, and the risk of life threatening complications of VBAC can be reduce with appropriate criteria (such as previous transverse incision, not using induction for delivery, noting the interval from the previous CS). Some studies proposed that 18 months interval between previous CS and VBAC are adequate (20). Bangal showed that uterine rupture happened in women attempted to have VBAC before 2 years interval from previous C-section (21). 

**Table 2:** compression of complication between successful and unsuccessful VBAC

	**Successful VBAC** **n (%)**	**Unsuccessful VBAC** **n (%)**	**p value**
Neonatal complications			0.002*
Yes	4 (57.1)	5 (6.8)	
No	3 (49.2)	68 (93.2)	
Post partum hemorrhage			0.761**
Yes	0	2 (6.4)	
No	7 (100)	73 (93.6)	
Successful Breast feeding			0.002**
Yes	3 (42.9)	68 (95.8)	
No	4 (57.1)	5 (6.8)	

* chi-square test;

** Fisher exact test

In the present study none of pregnant women died. Mone confirmed that VBAC was not associated with higher mortality rate (25). Damle showed that long term complications were less in VBAC group (22). These findings revealed the safety of VBAC for mothers.

6.8% of neonates in VBAC group needed resuscitation or NICU admission. This incidence was near to Blanchette findings (4.2%) (26). Our results showed that neonatal complications were higher in unsuccessful VBAC. This was confirmed previous studies findings (20, 23, and 25). Celeste reported that low Apgar score and NICU admission are more frequent in patients with VBAC failure (27). None of our neonates had transient tachypnea; Gilbert reported 2.7% transient tachypnea in neonates delivered by VBAC method (28). This might occur due to larger sample size in his study. In our study 95% of neonates were older than 37 gestational age, which can reduce the prevalence of respiratory complications in our study population. There are some evidences about the relation between the use of anesthetics and painkiller during CS and neonatal respiratory complications (22, 27). On the other hand, fetal distress might be the cause of VBAC failure, so neonatal complications are more common in this group. 

None of our neonates died in the perinatal period, like Bangal and Blanchette studies (20, 25). Phelan reported VBAC neonatal mortality rate 4.5 in 1000 live births (23). It seems that neonatal complications could be reduced effectively by focusing on the selection criteria for VBAC. 

Mean hospital admission duration was lower in VBAC (21). It would be a very important factor for decreasing nosocomial infections and long term complications. Shorter hospital stay promotes mother psychology status and also, reduces delivery expenses. 

95% of our patients could feed their children right after delivery. Regan reported breastfeeding in 95% of VBAC cases (13). Lower incidence f breastfeeding is expected on unsuccessful VBAC (CS) because of anesthesia and recovery time delayed the skin contact between mother and child, particularly in first hours after birth. 

One major limitation of our study was the absence of control group. On the other hand longer studies with larger sample size might result in more accurate findings. One other probable limitation of our study was its design; our project was conducted in academic hospitals in which supervision of experienced obstetricians can influence the incidence of maternal and neonatal complications.

## Conclusion

Our study revealed that VBAC is a safe method, if women have been selected with appropriate criteria. So, most cases with previous C-section with none-repeated indications have the chance for VBAC, particularly in centers with the emergency CSs facilities.
